# Cross Talk Between Macrophages and Cancer Cells in the Bone Metastatic Environment

**DOI:** 10.3389/fendo.2021.763846

**Published:** 2021-11-03

**Authors:** Lena Batoon, Laurie K. McCauley

**Affiliations:** ^1^ Department of Periodontics and Oral Medicine, University of Michigan School of Dentistry, Ann Arbor, MI, United States; ^2^ Bones and Immunology Group, Mater Research Institute, The University of Queensland, Brisbane, QLD, Australia

**Keywords:** macrophage, tumor microenvironment, skeletal metastasis, bone marrow, myeloid derived suppressor cells, efferocytosis, angiogenesis

## Abstract

The skeleton is a common site for cancer metastases with the bone microenvironment providing the appropriate conditions for cancer cell colonization. Once in bone, cancer cells effectively manipulate their microenvironment to support their growth and survival. Despite previous efforts to improve treatment modalities, skeletal metastases remain with poor prognoses. This warrants an improved understanding of the mechanisms leading to bone metastasis that will aid development of effective treatments. Macrophages in the tumor microenvironment are termed tumor associated macrophages (TAMs) and their crosstalk with cancer cells is critical in regulating tumorigenicity in multiple cancers. In bone metastases, this crosstalk is also being increasingly implicated but the specific signaling pathways remain incompletely understood. Here, we summarize the reported functions, interactions, and signaling of macrophages with cancer cells during the metastatic cascade to bone. Specifically, we review and discuss how these specific interactions impact macrophages and their profiles to promote tumor development. We also discuss the potential of targeting this crosstalk to inhibit disease progression. Finally, we identify the remaining knowledge gaps that will need to be addressed in order to fully consider therapeutic targeting to improve clinical outcomes in cancer patients.

## Burden of Bone Metastases

Bone metastases are common complications of solid tumors particularly in patients with cancers of the breast, prostate or lung (1, 2). Despite previous efforts to improve cancer diagnosis and treatment modalities, skeletal metastases remain with poor prognoses and high mortality and only about 10% one-year survival after bone metastasis diagnosis e.g. in lung cancer patients (3). Furthermore, such metastases result in considerable morbidity as they can cause limb dysfunction, impaired mobility, pathological fractures, spinal cord compression, and severe pain, significantly affecting patients’ quality of life (2, 4). Management of metastatic bone diseases imposes a huge burden on health care systems due to the substantially high costs associated with extensive use of medical resources (5, 6). Collectively, this warrants an improved understanding of the mechanisms leading to bone metastasis and progression that will aid development of effective treatments to prevent and alleviate the morbidity and mortality associated with bone metastases.

## Metastasis and the Bone Microenvironment

**Figure 1:**
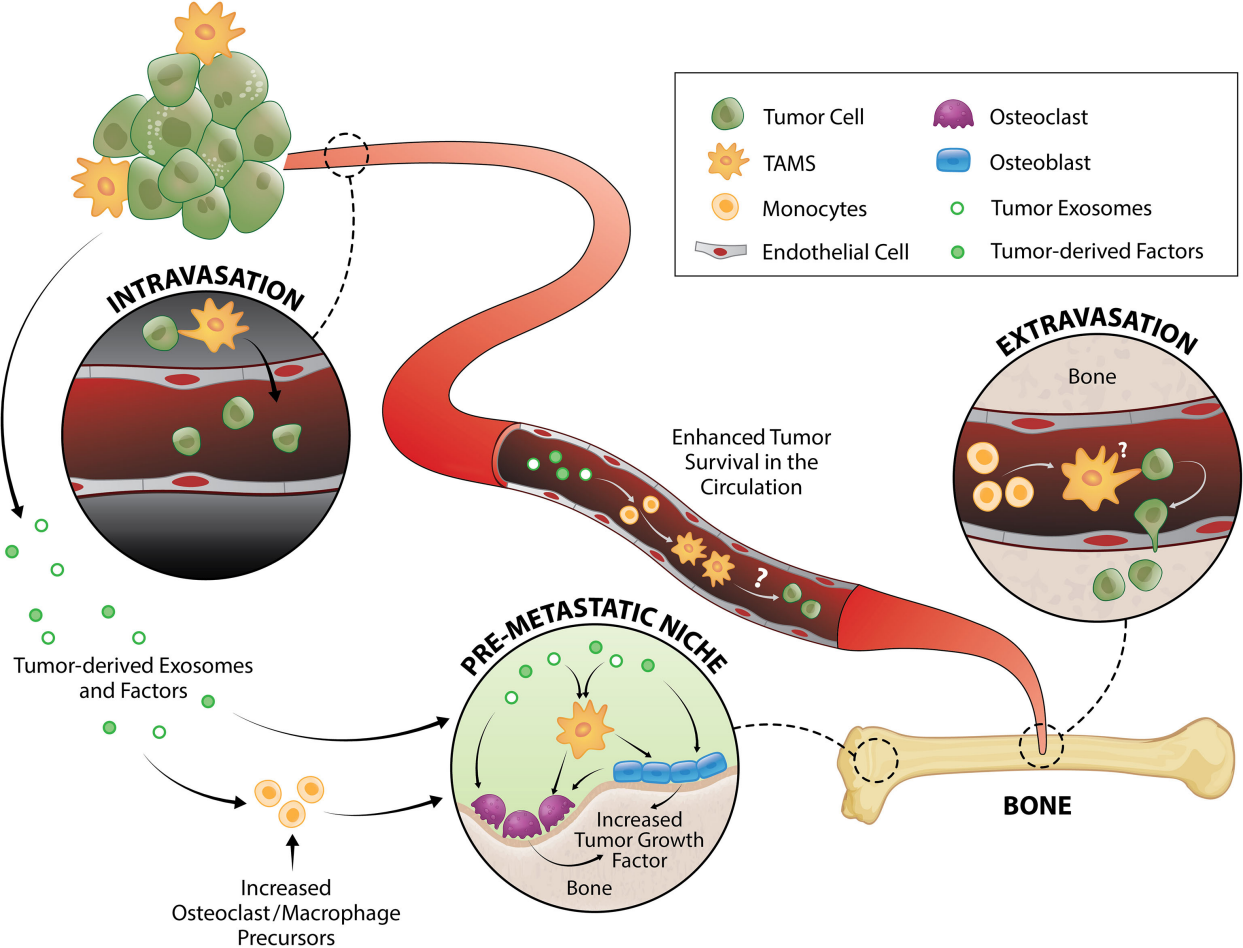
TAM and tumor crosstalk promotes the early metastatic cascade. TAMs contribute to tumor intravasation, extravasation and pre-metastatic niche formation. Direct interaction between TAMs and tumor cells induce migration from primary tumor site to the vessel wall and subsequently, tumors egress into the circulation where they generate fragments that recruit macrophages and monocytes required for successful early metastasis. Tumor-derived exosomes and factors also trigger pre-metastatic niche formation directly or by enhancing monocyte/macrophage recruitment that serves as osteoclast precursors or stimulate osteoblast function, both of which result to release of a myriad of tumor growth factors.

While cancer cell formation is a product of genetic/epigenetic aberrations in normal cells (7), development and progression requires corruption of the normal microenvironment by cancer cells to support their growth, survival and metastatic colonization. The metastatic process begins with *intravasation *of cancer cells from the primary site into the lymphatic or vascular system before *extravasation *of these disseminated tumor cells (DTCs) into compatible secondary sites that support their growth (8) (**Figure 1**). Some tumors, such as prostate (9), pancreatic (10), and oral squamous cell (11) cancers, have been shown to migrate along nerves (12) or the abluminal surface of the endothelium (13). The final stage of the metastatic cascade is colonization of the distant organ which is usually achieved through three key steps: colonization, dormancy and outgrowth (8). Despite bone being a common site of metastasis in addition to liver and lung, the exact mechanisms influencing metastatic progression to the skeleton remain unclear. In accordance with the ‘seed and soil’ hypothesis (14), it is widely accepted that the bone microenvironment provides an ideal niche allowing metastatic tumor cells to thrive.

The bone microenvironment is a highly dynamic compartment consisting of diverse cell types as well as an extracellular matrix with a copious array of cytokines. Cells within this microenvironment include the bona fide bone cells (osteoblasts, osteocytes and osteoclasts), hematopoietic and immune cells (notably macrophages, natural killer (NK) cells, B cells and T cells), stromal cells, adipocytes, fibroblasts and endothelial cells (15). Interactions between these cells regulate multiple physiological processes including hematopoiesis, bone remodeling as well as bone and bone marrow homeostasis. Once in bone, cancer cells secrete cytokines and growth factors to interact with these cells, hijacking their normal functions to provide tumor growth requirements. This creates a pathological crosstalk causing lesions that are either osteoblastic (increased bone formation), osteolytic (bone destruction) or mixed (16, 17) depending on the primary mechanism of interference with normal bone remodeling. There is mounting evidence demonstrating that interaction between tumor cells and the supportive stroma plays a crucial role in development and progression of bone metastasis (18–22).

## Macrophages – Key Cells in the Bone and Tumor Microenvironment

Macrophages are abundant immune cells in the bone microenvironment. They are heterogeneous myeloid cells (23) expressing diverse and adaptive transcriptomes (24, 25), and thus, are capable of rapidly responding to environmental changes (26). Macrophages are present within virtually all tissues (27) throughout life (termed “tissue resident macrophages”) contributing to tissue homeostasis, tissue specific physiology and innate immune surveillance. They can be replenished by the circulating monocyte pool (28) but some are established during embryonic development, persist into adulthood and have the ability to self-renew (29–31). Both monocyte-derived (32–36) and tissue-resident embryonic-derived (36, 37) macrophages have been shown to contribute to tumor development. Three subsets of tissue resident macrophages in the bone have been characterized; erythroblastic island macrophages (EIM), hematopoietic stem cell (HSC) niche macrophages and osteal macrophages (osteomacs). EIM and HSC niche macrophages are located in the bone marrow (38) while osteomacs are present on human and mouse osteal tissues (39).

According to the conventional binary classification, upon tissue insults, various signals may “activate” macrophages into either classically activated pro-inflammatory “M1” [induced by interferon- gamma (IFN-γ), lipopolysaccharide (LPS), tumor-necrosis factor-alpha (TNF-α)] or alternatively activated anti-inflammatory “M2” (40). Based on *in vitro* studies, M2 macrophages can be subdivided into four distinct subtypes depending on the nature of inducing agent and the expressed markers: M2a, M2b, M2c and M2d (41, 42). However, whether these subtypes occur *in vivo* remains unclear. It should also be noted that the actual activation state of macrophages is much more complex than the M1 and M2 classification – a concept which originated from the significant biases in macrophage polarization between C57Bl/6 and BALB/c mice (43, 44). Macrophages from various mouse strains differ markedly in their gene expression profiles (45, 46) however these differences were not known at the time the concept was proposed. Furthermore, the M1/M2 nomenclature is not well-supported by large-scale transcriptomic data which instead favors a broad spectrum of activation states (47, 48). Hence for the remainder of this review, we will use “M1-like” and “M2-like” to refer to these conventional activation states rather than M1 or M2 alone which are considered over-simplified.

Macrophages are a major component of the tumor microenvironment, representing 50% of the tumor mass (49). The original and early hypotheses proposed that macrophages are involved in antitumor immunity, however there is substantial clinical and experimental evidence that in majority of cases, macrophages in the tumor microenvironment, termed tumor-associated macrophages (TAMs), enhance progression to malignancy. Once cancers leave the primary site, monocyte-derived macrophages are recruited to support the metastatic cascade. These macrophages have been termed metastasis-associated macrophages (MAMs). Primary (50) and review (51–53) articles have acknowledged the existence of both macrophage types suggesting differences to be based on origin with TAMs arising from resident macrophages at the primary tumor site and MAMs differentiating from inflammatory monocytes/macrophages at the metastatic site. However, this idea is challenged by recent evidence demonstrating that TAMs can be sourced from both monocytes and resident macrophages (36, 37, 54, 55). Furthermore, while TAMs and MAMs have been proposed to be distinct, for example due to different fibroblast activation protein alpha (FAP) expression (56), molecular markers that fully differentiate TAMs and MAMs are still lacking. Therefore, further studies are required to better understand the similar/distinct phenotypes of TAMs and MAMs and to clarify literature inconsistencies, although it is likely that their molecular signatures vary across different cancer types.

It is generally believed that M1-like macrophages have anti-tumor activity while M2-like macrophages promote tumor progression. In many types of human cancers, the density of macrophages is strongly correlated with poor prognosis (57–61) particularly those with M2-like polarization (60, 62–64). Macrophage-derived exosomes have also been shown to facilitate tumor metastasis and development (65–67). Furthermore, macrophage proliferation, differentiation and survival are regulated by colony stimulating factor 1 (CSF1) receptor (CSF1R) which can be activated by two ligands: CSF1 and interleukin (IL)-34 both of which have been associated with poor patient outcomes. For example, overexpression of CSF1 in breast (68, 69), prostate (70), pancreatic (71), hepatocellular (72) and colorectal (73) cancers, and IL-34 expression in hepatocellular carcinoma (74), lung (75) and colorectal (76) cancers are associated with disease progression and unfavorable prognosis. Notably, serum levels of CSF1 are increased in prostate cancer patients with bone metastasis (70). Consequently, targeting macrophages and their tumor-promoting functions are major areas of research in the pursuit of successful therapy (77).

## Macrophages and Cancer Crosstalk in Bone Metastasis Progression

### Intravasation and Extravasation

Intravasation, and therefore generation of circulating tumor cells (CTCs), is one the first key steps in the development of distant metastases. This process can be separated into two stages: 1) invasive migration of tumor cells through the extracellular matrix to the vessel wall, and 2) penetration through the vessel wall. Two TAM subsets have been identified that support these processes: migratory macrophages guide cancer cells toward blood vessels and perivascular macrophages assist their entry into the circulation (**Figure 1**). A recent study showed that C-C motif chemokine receptor type 2 (CCR2)-recruited monocytes differentiate into migratory TAMs that assist with cancer cell motility through the extracellular matrix before differentiating into perivascular macrophages *via* C-X-C motif chemokine receptor 4 (CXCR4)/CXCL12 signaling (78). CXCR4/CXCL12 has long been known as a key signaling pathway in breast and prostate cancer metastasis to bone (79, 80). *In vitro* (81, 82) and *in vivo* (82) imaging demonstrated that direct contact between macrophages and cancer cells facilitate cancer cell migration from the primary tumor site. Upon physical contact, macrophages induce RhoA signaling in tumor cells to form invadopodia (82) - actin-rich membrane protrusions that degrade the extracellular matrix. This interaction is further promoted by a positive feedback loop whereby cancer cells produce CSF1 and TAMs express epidermal growth factor (EGF) (81, 83) which is a positive regulator of RhoA signaling (84, 85). Once in the blood vessel, vascular endothelial growth factor A (VEGFA) signaling from TIE2^+^ perivascular macrophages (86, 87) causes vascular leakiness, allowing tumor cell egress.

CTCs travel as single cells or in clusters (88) in the circulation where they are exposed to various insults including immune destruction and mechanical forces due to fluid shear stress. Very little is known about the survival mechanisms that CTCs employ while in the circulation, but putative mechanisms have been proposed and reviewed elsewhere (89–91). Using an intravital two-photon lung imaging model in mice, it has been shown that CTCs generate fragments that serve as immune-interacting intermediates to recruit phagocytic myeloid cells that were predominantly macrophages and monocytes for successful early metastasis (92) (**Figure 1**). Although CTCs use various strategies to survive in the circulation, their metastatic potential ultimately depends on rapid extravasation into another tissue. Cancer cell extravasation appears to be one of the least studied steps in the metastatic cascade perhaps due to the difficulty in investigating this event that occurs well hidden within intact organs. As CTCs encounter capillary beds, where extravasation typically occurs (93, 94), they undergo physical arrest and are thought to form adhesive interactions with their surface receptors to the complementary sites on the endothelium (95, 96). CTCs then transmigrate *via* paracellular migration where they extend projections between adjacent endothelial cells into the extravascular space before moving their cell body, nucleus, and trailing edge across the endothelium (93, 97–99). In the bone marrow, the sinusoidal capillaries are lined with endothelial cells and discontinuous basal lamina (100) which may facilitate simpler extravasation and therefore contribute to high metastatic incidence in bone (1, 2).

MAMs have been implicated in breast cancer cell extravasation in the lung (50, 101). This population originated from circulating monocytes and is characterized as F4/80^low^CD11b^high^Ly6C^low^ macrophage population (50, 102). While the specific mechanism underlying the macrophage contribution in CTC extravasation is not fully understood, it has been shown that direct tumor cell-macrophage interaction is required for this process (101) (**Figure 1**). Of note, elevated peripheral blood monocytes (macrophage precursors) have been associated with poorer disease prognosis in several cancer types (103–108), which could be due to monocytes serving as MAMs precursors. Furthermore, overexpression of the monocyte chemoattractant protein-1 (MCP-1, also known as CC chemokine ligand 2/CCL2 which is the ligand for CCR2) in human prostate cancer cells increases macrophage accumulation and enhances bone metastasis (109). In breast cancer bone metastasis, MAMs are largely derived from Ly6C^+^CCR2^+^ monocytes and they express high levels of CD204 and IL4R (35). While it was shown that these MAMs promote bone metastasis in an IL4R-dependent manner (35), it is unknown whether they are directly involved in extravasation to bone or other steps in the metastatic cascade.

### Colonization, Dormancy and Outgrowth

Bone marrow-derived macrophages (BMDMs) have been implicated in tumor growth and metastasis such as in metastatic lung cancer (110, 111). Using a murine model, Cho et al. demonstrated that co-injection with mammary carcinoma cells with bone marrow-derived M2-like macrophages promoted tumor growth and lung metastasis (110). Given the bone and its marrow are abundant in resident macrophages, it is likely that they provide an attractive microenvironment for metastases because bone and bone marrow macrophages can largely contribute to formation of a pro-tumorigenic niche.

#### Pre-Metastatic Niche Formation

It is now being increasingly recognized that organs of future metastasis are not passive receivers of CTCs; instead, they have been primed and actively modified to support incoming cancer cell needs even prior to metastatic spread. These primed sites are termed “pre-metastatic niches” and they form as a result of tumor-secreted factors and tumor-shed extracellular vesicles (112) (**Figure 1**). Osteoclasts have been implicated in pre-metastatic niche formation for bone metastases. They are myeloid-derived cells commonly known as the bone-resorbing cells. While some still regard osteoclasts as a resident macrophage in bone, *in vivo* evidence support that osteomacs and osteoclasts are distinct mature myeloid cell types (113, 114). In metastatic breast cancer, tumors secrete lysyl oxidase (LOX) that triggers osteoclastic bone resorption resulting to formation of pre-metastatic lesions (115, 116) as well as release of bone-matrix stored growth factors including insulin-like growth factors (IGFs), transforming growth factor-β (TGFβ), fibroblast growth factor (FGFs), and bone morphogenetic proteins (BMPs) which all have pro-tumorigenic effects (117–119). Recently, Yuan et al. also demonstrated that breast cancer exosomes contribute to pre-metastatic niche formation in the bone by transferring exosomal miR-21 to osteoclasts (120) which triggers their activation and survival (121). Osteoclastogenesis in metastatic bone cancer has been suggested to be mediated by CD137L-CD137 signaling pathway where CD137 enhances monocyte/macrophage migration and differentiation to osteoclasts (122) (**Figure 1**).

#### Immunosuppression

**Figure 2:**
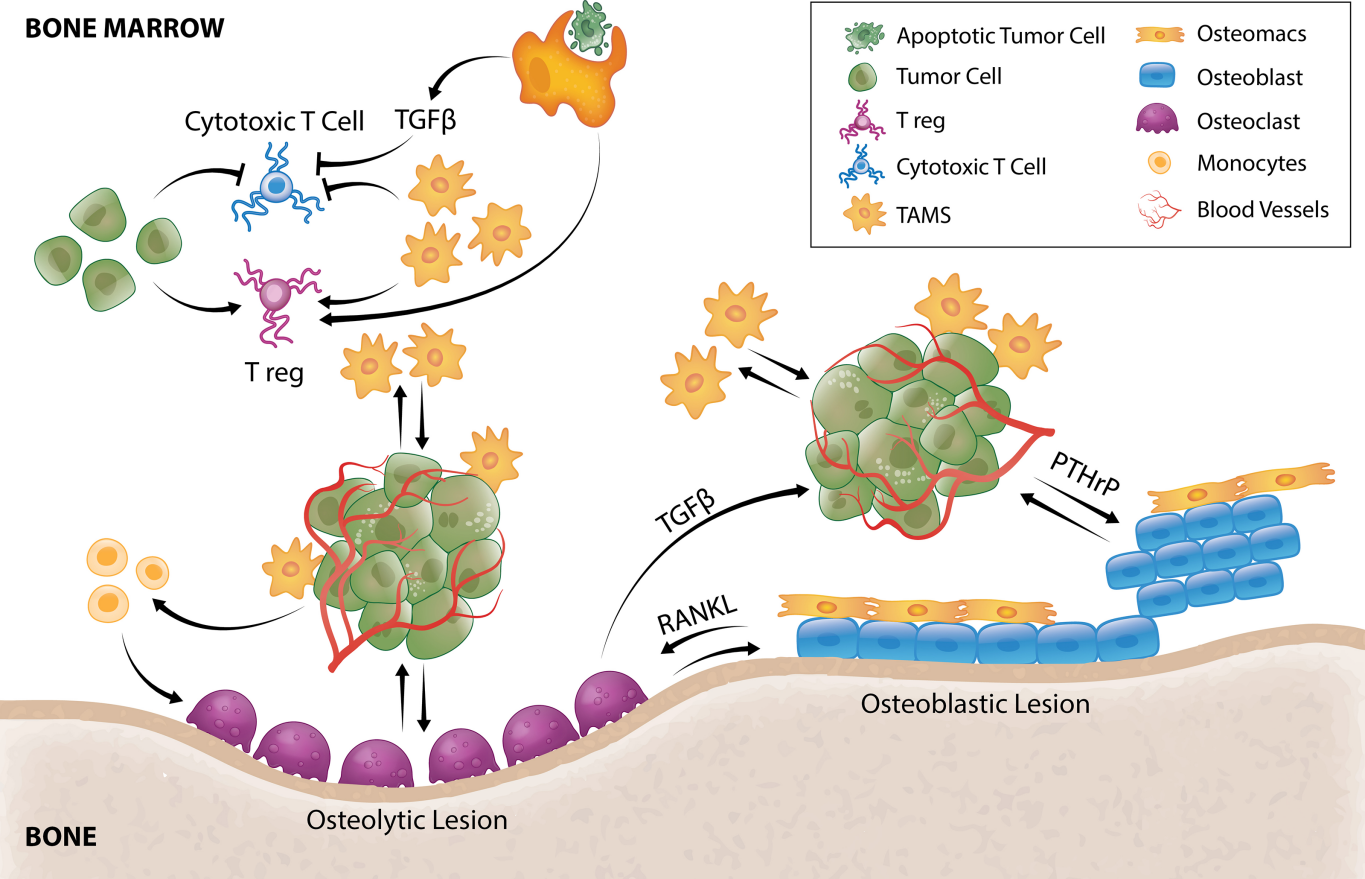
TAM and tumor crosstalk promotes tumor survival and outgrowth in bone. Efferocytosis of apoptotic tumor cells by TAMs results in TGFβ secretion that inhibits cytotoxic T cells and stimulates Tregs. TAMs and tumor cells can also directly hijack immune checkpoint pathways to negatively regulate T-cell anti-tumor function. TAMs secrete a multitude of pro-angiogenic factors that induce tumor angiogenesis allowing delivery of oxygen, nutrients and growth factors. Tumor cells can directly stimulate pro-tumorigenic osteoblastic or osteolytic lesion formation and/or recruit TAMs or osteoclast precursors to support these processes. In osteolytic lesions, tumors secrete factors that directly stimulate osteoclastic-mediated resorption to release bone-derived tumor growth factors or stimulate osteoclastogenesis by promoting osteoblast-derived RANKL. Osteoclastogenesis also releases TGFβ from the bone matrix that directly stimulates tumor cells to secrete PTHrP that can enhance osteoblasts function to form osteoblastic lesions or enhance osteoblast production of RANKL to form osteolytic lesions. Osteomacs which are abundant within osteoblastic lesions also support osteoblast-mediated bone anabolism, suggesting that they could be a key driver of tumor-induced bone formation.

After arriving in the bone microenvironment, cancer cell survival is determined by their ability to resist immunity and other bone tissue defenses and settle within the specialized local niches. Newly disseminated cancer cells are particularly vulnerable to immune surveillance by macrophages and T cells (**Figure 2**). In a colorectal cancer model, TAMs have been shown to be pivotal constructors of tumor collagenous extracellular matrix, upregulating synthesis and assembly of collagens (collagen types 1, VI, XIV), and instructing their deposition in areas of tumor development (32). Coordinated tumor and tumor-stromal cell interactions subsequently remodel these extracellular matrix components forming a physical barrier to evade immune surveillance (123).

The phagocytic function of macrophages plays an essential role in bone homeostasis (124) where they clear pathogens, debris as well as apoptotic cells (efferocytosis). Under normal physiology, efferocytosis serves as a waste disposal mechanism but also a pro-resolving phenotype in macrophages (125). In bone metastasis, efferocytosis of apoptotic cancer cells *via* milk fat globule-EGF facto 8 (MFG-E8) bridge protein induces an M2-like phenotype in TAMs (126) which has a well-documented crucial role in immunosuppression including influencing T cell recruitment and function (127). Efferocytic M2-like macrophages under normal circumstances (128, 129) and M2-like TAMs in cancer setting (130) have been shown to secrete TGF-β1 which can, 1) directly inhibit cytotoxic T cell (CTL) expression of cytolytic gene products (perforin, granzyme A, granzyme B, Fas ligand, IFN-γ) required for cytotoxicity (131) or, 2) act indirectly by stimulating differentiation of regulatory T cells (Tregs) (132) (**Figure 2**). *In vitro*, macrophage efferocytosis of prostate cancer cells induced expression of inflammatory cytokines including CCL5, CXCL1, CXCL5 and IL-6 (133) which can orchestrate growth of bone metastases (133–137). In particular, CXCL5 has been shown to accelerate growth of metastatic prostate cancer in bone (133).

Tumor cells are also well-known to hijack the programmed cell death-1 (PD-1)/PD-1 ligand (PD-L1) pathway to escape immunosurveillance (138). In fact, it is one of the best-studied and most promising immune checkpoint drug targets with benefits reported in different cancer types (139), although bone metastases appear to impair its efficacy (140). PD-1 is an immune checkpoint receptor expressed by activated T cells while tumor cells frequently express PD-L1. PD-1/PD-L1 engagement has been shown to negatively affect CD8^+^ T cell activity (141), allowing tumors to escape T cell-mediated cell death. TAMs also express high levels of PD-L1 in different types of cancers (142–145), indicating that they could also directly suppress T cell cytotoxic functions which is reflected by improved anti-tumor T cell activity following macrophage depletion (145–147). Recently, Gordon et al. showed that TAMs from human colorectal cancer samples and colon cancer cell line CT26 also express high levels of PD-1 (148). PD-1 expression in TAMs negatively correlated with phagocytic potency against tumor cells while blockade of PD-1/PD-L1 improved phagocytosis and reduced tumor growth (148). Together, these suggest that the PD-1/PD-L1 pathway has a significant role in TAM function and tumor survival.

Another immune checkpoint pathway that could negatively regulate T-cell anti-tumor function in the setting of cancer is cytotoxic T-lymphocyte–associated antigen 4 (CTLA-4) and macrophage Clever-1. CTLA-4 upregulation and binding with CD80/CD86 promotes T cell anergy (149), and cancer cells (150–152) and TAMs (153) have been shown to express CD80 and CD86. The macrophage Clever-1, a multifunctional adhesion and scavenger receptor expressed by immunosuppressive macrophages and TAMs, has also been shown to have inhibitory effect on CD8^+^ T cells (154). Viitala et al. showed that genetic deficiency of macrophage Clever-1 or its blockage resulted in activation of CD8^+^ T cells and impaired tumor growth (154). Interestingly, these immunostimulatory effects were comparable with PD-1 checkpoint inhibition (154). Overall, cancer cells can either suppress anti-tumor immune responses directly or indirectly by employing other cells such as TAMs (**Figure 2**).

#### Dormancy and Reactivation

The location of DTCs in the bone could be the key to their fate. Tumor cells delivered to the bone remodeling compartments (metaphyseal trabecular bone regions) will be exposed to a microenvironment rich in growth factors that promote growth and survival and thus, they may proliferate immediately (155). Conversely, those arriving in the quiescent region (endosteal surface) will encounter a microenvironment that promotes tumor cell dormancy (155). Given that the inactive endosteal surface predominates (156), it is conceivable that DTCs mainly undergo dormancy once arrested in bone. Indeed, DTCs are detected in the bone marrow of breast (157–159) or prostate (160) cancer patients that neither correlated with tumor stage nor size which could indicate tumor cells have entered dormancy. Similarly, DTCs have been detected in bone marrow aspirates of patients with ovarian and endometrial cancer that did not correlate with established clinicopathological factors (161). Moreover, the metaphyseal trabecular region of long bones is normoxic while the diaphyseal region is more hypoxic (162) and hypoxia regulates key tumor dormancy factors (163). Hypoxia also supports macrophage recruitment *via* hypoxic tumor-derived cytokines (164, 165) and modifies polarization of macrophages, selectively promoting M2-like phenotype in multiple cancers (166–169) through activation of ERK signaling (166).

Recently, an *in vitro* study showed that macrophage exosomes regulate dormancy of breast cancer cells in bone marrow stroma (170). Exosomes from M2-like macrophages were shown to sustain quiescence and reduce proliferation of cancer cells while M1-like macrophage-derived exosomes reversed dormancy by NFκB activation (170). In mice with subcutaneous tumors and patients with non-small cell lung cancer (NSCLC), TAMs have been shown to exacerbate tumor hypoxia *via* AMP-activated protein kinase (AMPK) and peroxisome proliferator-activated receptor gamma coactivator 1-alpha (PGC1α) activation (171) which could perpetuate tumor dormancy.

Tumor dormancy can last for years and decades, but what is perhaps the most clinically relevant phase is the exit from dormancy, also termed the “reactivation” phase, that requires escape from the dormant state to proliferate actively and form micrometastases. This process is nursed by the surrounding supportive niches (172). The osteoblastic niche has been shown to play an important role in controlling tumor cell dormancy (173, 174) through the Wnt5a/ROR2/SIAH2 signaling axis (174) while remodeling of the endosteal niche by osteoclasts have been shown to induce reactivation (173). Data is lacking that directly demonstrates the role of TAMs in tumor reactivation in bone metastasis setting. However, this would not be surprising given it has been shown that: 1) the tumor dormancy-reactivation process is reversible (173), 2) TAMs provide or stimulate tumor dormancy signals, and 3) inhibition of dormancy signals reawakens dormant cancer cells (175). Of note, tumor-derived vascular cell adhesion molecule 1 (VCAM-1) has been shown to promote transition from indolence to overt metastasis by recruiting CD11b^+^ monocytes and increasing osteoclast activity (176). Recently, abscisic acid was reported to regulate dormancy of prostate cancer disseminated tumor cells in the bone marrow (177) and separately studies identified abscisic acid as an inducer of the M1 phenotype (178). Further studies that investigate the contribution of TAMs in dormancy and tumor reactivation are warranted.

#### Angiogenesis

Following reactivation, the final step of metastasis occurs when DTCs proliferate, becoming independent of the microenvironment and ultimately modifying it to support outgrowth. While a hypoxic environment induces dormancy, angiogenesis is required for tumor proliferation as it allows delivery of oxygen, nutrients and growth factors. For decades, macrophages have been implicated in neovascularization of tumors (179). In healthy tissues, blood vessels are in a quiescent state and angiogenesis is only transiently activated in response to certain stimuli. Conversely, in tumor progression, an “angiogenic switch” results with continuous sprouting of *de novo* vessels. The tumor vasculature differs from a normal vascular network as it is characterized by hyperpermeability, excessive and convoluted branching and erratic blood flow (180). TAMs are important for this angiogenic switch as they represent a potent source of a multitude of other pro-angiogenic factors including those from the EF-hand calcium-binding cytosolic (S100A) protein family, semaphorins family and chitinase-like proteins (181). They have also been shown to promote angiogenesis in human tumors (182, 183) and in animal models of breast (184) and prostate (185) cancers. Tumor cells, under hypoxic conditions, have been proposed to produce oncometabolites including lactate and succinate that induce a pro-angiogenetic phenotype in TAMs (186). M2-like TAMs secrete VEGF-A (183) which is considered a major mediator of tumor angiogenesis (187) and an indicator of metastatic potential to bone in malignant prostate cancer (188). Conversely, repolarization of TAMs towards an M1-like phenotype leads to tumor vessel normalization (189, 190). For new capillaries to sprout, degradation of the host vessel at specific sites needs to occur and this process is mediated by proteinases such as matrix metalloproteinases (MMPs). In some tumors, TAMs have appeared to be a major source of MMP9 which mediates extracellular matrix degradation and releases VEGF-A from the extracellular matrix reservoir (18, 191–193). The release of VEGF-A can then induce a positive feedback loop on angiogenesis by further recruiting pro-angiogenic and immunosuppressive macrophages through their VEGF receptor (VEGFR1) (194, 195).

#### Osteoblastic and Osteolytic Bone Lesions

Significant effort has been made into characterizing the mechanisms associated with overt tumor growth in the skeleton – a paradigm commonly referred to as the “vicious cycle” (196). Herein, tumor cells can stimulate excessive bone formation (osteoblastic) or resorption (osteolytic) leading to disruption of bone integrity and production of factors that fuel cancer proliferation which then results to further bone formation or destruction (118). Osteolytic bone lesions are usually characteristic of the majority of breast cancers and non-small cell lung cancer while osteoblastic lesions are common in prostate and small cell lung cancers (197). Of note, in breast (198) and prostate (199) cancer patients, both type of lesions can be present. Tumor cells can instigate bone formation by producing osteogenic factors (**Figure 2**) including BMPs, EGFs, endothelin-1 and platelet derived growth factor (PDGF) (200). In turn, activated osteoblasts can produce pro-tumorigenic factors (**Figure 2**) including IL-6, CCL2 and VEGF (201). Prostate cancer cell exosomes have been shown to stimulate bone formation by inhibiting osteoclast fusion and differentiation (202) causing remodeling imbalance that ultimately results in net bone formation. Prostate cancer cells also produce CCL2 to recruit macrophages/TAMs or osteoclasts (109) that assist with pro-tumorigenic lesion formation. *In vitro*, bone marrow macrophages and prostate tumor cell interaction upregulates cathepsin K expression in macrophages which promotes tumor progression in bone (203).

CD68^+^ TAMs are present within patients’ prostate cancer skeletal lesions where they are directly associated with woven bone (204). Similarly, in a mouse model of prostate cancer bone metastasis, F4/80^+^ macrophages were abundant within the skeletal lesions and osteomacs were directly associated with *de novo* pathological bone (204). Osteomacs, including their efferocytic function, support osteoblast-mediated bone anabolism (113), suggesting that they could be a key driver of tumor-induced bone formation. This idea is strongly supported by the significant reduction in pathological woven bone deposition when CD169^+^ osteomacs/TAMs were depleted (204). Nonetheless, further investigation into the specific molecular interaction between osteoblasts and osteomacs are required to understand how these pathways are modulated in the tumor setting.

In metastatic cancers with osteolytic lesions, tumors secrete factors such as IL-8, IL-11, CSF1 and TNFα which directly stimulate osteoclastic-mediated resorption (**Figure 2**) to release bone-derived tumor growth factors (117). Recently, tumor-derived monoamine oxidase A (MAOA) was also shown to stimulate osteoclastogenesis by promoting osteoblast-derived receptor activator nuclear kappa B ligand (RANKL) and IL6 expression (22). As a result of osteoclastic bone resorption, TGFβ is released from the bone matrix and directly stimulates tumor cells to secrete parathyroid hormone–related protein (PTHrP) (205) (**Figure 2**). PTHrP has dual effects on bone remodeling. It stimulates osteoblasts which can result in osteoblastic lesion formation (206, 207) but it is also a potent stimulator of osteoclastogenesis by enhancing osteoblast production of RANKL (**Figure 2**) and CCL2 (208, 209). There is currently very limited literature on the contribution of TAMs in the formation of osteolytic lesions, but CD68^+^ macrophages have been reported within osteolytic lesions of prostate cancer patients (210). Furthermore, Movila et al. demonstrated that macrophage migration inhibitory factor (MIF) mainly produced by macrophages within the bone lytic site acts as a chemoattractant to osteoclast precursors, leading to further recruitment of osteoclasts to sustain bone destruction (211).

## Therapeutically Targeting Macrophage and Cancer Crosstalk in Bone Metastases

**Figure 3:**
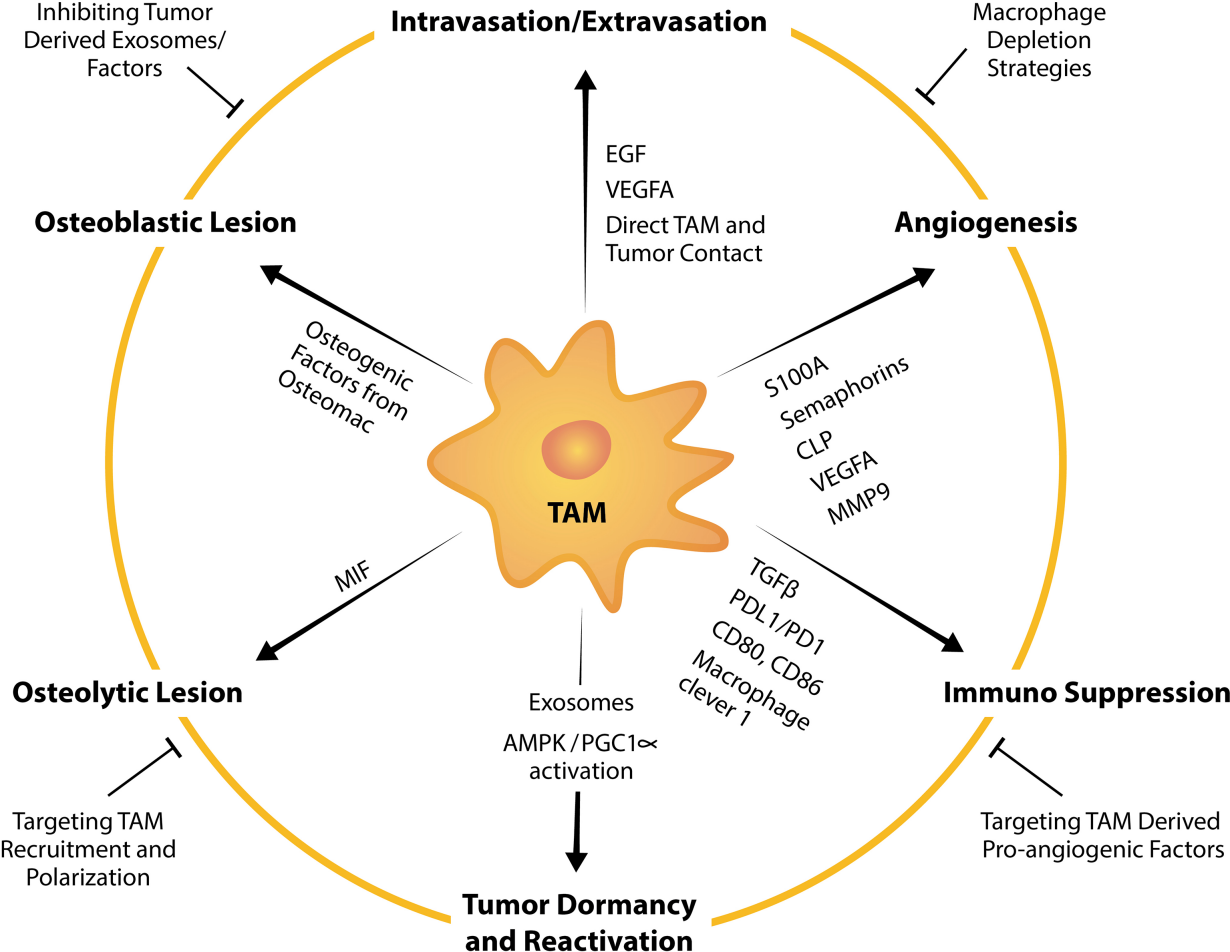
TAM factors and markers that facilitate tumor bone metastasis and strategies to suppress TAM-mediated promotion of metastasis. TAMs influence tumor intravasation, extravasation, angiogenesis, dormancy, reactivation, formation of osteolytic and osteoblastic lesions, and immunosuppression by expressing or releasing pro-tumorigenic factors. For example, TAMs are a potent source of a multitude of pro-angiogenic factors including VEGFA, MMP9 and those from the EF-hand calcium-binding cytosolic (S100A) protein family, semaphorins family and chitinase-like proteins. Strategies to target TAM pro-tumorigenic functions summarized in this schematic include broad macrophage depletion strategies, targeting pro-angiogenic factors from TAMs, inhibiting TAM recruitment, TAM repolarization and inhibiting tumor-derived exosomes or factors that instigate TAM and tumor pathological crosstalk.

Bone metastases remain incurable and current management are focused on minimizing pain, resolving or minimizing the risk of developing skeletal related events (SREs) and inhibiting tumor progression. Current treatment usually includes palliative radiotherapy (212) and systemic chemotherapy which directly targets malignant cells. Bone metastasis harbors resistance to chemotherapeutic drugs perhaps contributed to by bone being less perfused than other organs and thus, drugs which are administered intravenously do not reach the site in sufficient doses. In addition, pre-clinical work suggests that chemotherapies could condition the microenvironment *via* macrophage influx to be more receptive to metastasis (213). Efforts have been made in the utilization of bone-targeted nanoparticles loaded with anti-cancer drug which have demonstrated potential in inhibiting bone metastases (214–216). Extensive research aimed at understanding the “vicious cycle” that occurs in metastatic bone diseases has also led to approval of drugs that target the remodeling imbalance including bisphosphonates and the RANKL inhibitor denosumab, both of which target osteoclast function (217). However, while these drugs improve patients’ quality of life by reducing pain, fractures and inhibiting development of new lesions (218–220), metastatic bone cancer still progresses and thus, they have little or no benefit in overall survival (218–221). Therefore, management of bone metastases remains a significant clinical challenge and effective treatments are still an unmet clinical need. TAMs are involved in all steps of the metastatic cascade to the skeleton and have tumor-permissive and immunosuppressive characteristics. Therefore, targeting TAMs and their pro-tumorigenic functions has a promising potential in anti-cancer therapy (222) (**Figure 3**).

### TAM Depletion Strategies

Targeting the CSF1/CSF1R inhibitors has gained the most attention in this context with various approaches currently under clinical development for treatment of several cancer types including advanced castration-resistant prostate cancer (CRPC) with bone metastases (223). Most of the agents targeting the CSF1/CSF1R signaling axis are CSF1R inhibitors (224) with only two current clinical-stage programs targeting CSF1 and none targeting IL-34 thus far (223). Emerging data on the tolerability of CSF1/CSF1R-targeting agents indicate a relatively safe profile, with some studies reporting no dose-limiting toxicities (225–227). CSF1/CSF1R inhibitors have been shown to deplete TAMs (224, 226, 228) and reduce M2-like macrophage recruitment (229, 230). While these agents are not under clinical trial for metastatic bone disease other than advanced CRPC (NCT01499043), preclinical outcomes have been promising especially given CSF1R inhibitors have an additional benefit of targeting osteoclasts (231, 232). In a mouse model of metastatic lung cancer with osteolytic bone lesions, knockdown of CSF1 reduced the incidence of bone metastases (233). Similarly, in a rat model of mammary adenocarcinoma (234) and mouse models of breast cancer (232, 235, 236), treatment with CSF1R inhibitors prevented bone metastases and formation of osteolytic lesions. CSF1R targeting has also been demonstrated to successfully abrogate TAM infiltration and thus disrupt tumor promotion in animal models of prostate cancer (237, 238), however, whether these drugs have a role in inhibiting osteoblastic lesion formation remains underexplored. Overall, targeting the CSF1-CSF1R axis appear to be a promising strategy, however, it would be problematic to systemically deplete macrophages for a long period and thus, a more targeted approach would be ideal.

An example of a more directed approach are bisphosphonates which are bone-targeted drugs routinely used in metastatic disease with bone involvement to prevent or delay SREs and improve quality of life (219, 239). Given they effectively inhibit osteoclasts (240), bisphosphonates could have direct effect on preventing premetastatic niche formation and tumor proliferation by interfering with the “vicious cycle”. Interestingly, real-time intravital imaging has shown that bisphosphonates in extra-skeletal tumors are mainly taken up by TAMs (241). Moreover, bisphosphonates have been shown to inhibit macrophage proliferation and induce apoptosis *in vitro* (242, 243). Such effect on TAMs/macrophages could therefore contribute to the anti-tumor effects of these drugs.

The most investigated bisphosphonate to target TAMs in preclinical studies is the encapsulated clodronate (clodronate liposomes) which are preferentially taken up by macrophages owing to their phagocytic activities. In fact, clodronate (BONEFOS^®^) is approved for use in treatment of tumor-induced osteolysis in 67 counties though it remains commercially underdeveloped in the United States. This strategy has shown promise in inhibiting bone metastases in animal models of metastatic lung (243, 244), prostate (204) and breast cancers (245). In metastatic liver cancer model, macrophage depletion by clodronate liposomes significantly inhibited tumor progression, angiogenesis and lung metastasis (246). In mouse models of breast cancer, Zoledronic acid, another type of bisphosphonate, was shown to reduce TAM number and repolarized TAMs to M1-like anti-tumoral phenotype, reduce mammary carcinogenesis (247) and skeletal metastases (245). While these preclinical studies were promising, the clinical benefit of bisphosphonates is still limited to managing skeletal complications without improving patient’s overall survival. Current efforts are focused on utilizing bisphosphonates for a more targeted delivery of anti-cancer drugs to bone. While most studies on bisphosphonate-drug conjugates have been conducted *in vitro*, preliminary results are promising and reviewed elsewhere (248).

### Targeting TAM Recruitment and Polarization

Targeting the CCL2-CCR2 axis has also been attractive given its role in TAM recruitment. CCL2 promotes bone metastasis in experimental models of prostate cancer (249, 250) while its inhibition hinders TAM recruitment (251, 252) and correlates with reduced tumor burden (253). In *in vivo* models of metastatic prostate cancer, treatment with neutralizing anti-CCL2 antibodies either reduced systemic tumor burden including bone lesions (254) or completely inhibit bone metastases (255). CCL2 is increased by treatment with the chemotherapeutic drug docetaxel and protects prostate cancer cells from docetaxel-induced toxicity (256) but when combined with CCL2 blockade, docetaxel had striking impact on tumor suppression (254, 255). This anti-tumor efficacy demonstrated in preclinical studies were preceded by establishment of clinical trials in solid and metastatic cancers (257), however, the outcomes have somewhat been disappointing. The anti-CCL2 monoclonal antibody carlumab failed to inhibit tumor growth in early-stage clinical trials in prostate cancer as it was unable to sustain CCL2 blockade due to induction of compensatory mechanisms (258). A humanized neutralizing anti-CCR2 monoclonal antibody also went through a clinical trial for treatment of bone metastasis from solid tumors (NCT01015560). While the treatment was well-tolerated, only 14 out of 43 patients had considerable reduction in urine *n*-telopeptide, a biomarker of bone turnover rate, and the anti-tumor outcomes have not been disclosed despite study completion. Therefore, there is not enough evidence to support efficacy of CCL2/CCR2 targeting in skeletal metastases. Of note, interruption of CCL2 inhibition in models of metastatic breast cancer accelerated metastases and death (259). This highlights our incomplete understanding of the CCL2-CCR2 signaling network and that caution should be taken when considering targeting this axis in metastatic diseases.

Macrophages are extremely plastic; they can have pro-tumoral characteristics or be tumoricidal. This indicate that strategies which indiscriminately target TAMs/macrophages might only be partially effective, could induce undesirable side effects and long-term toxicities. Reprogramming TAMs could therefore be a more efficacious approach, providing a strategy where pro-tumoral macrophages can be re-educated towards an anti-tumoral phenotype to create a microenvironment that reject rather than nurture tumor cells. There are several strategies currently used to reprogram TAMs to be anti-tumorigenic in preclinical and clinical investigation including toll-like receptor agonists and monoclonal antibodies that induce TAM anti-tumor effects (260), and Hu et al. have recently identified many more (261). Trabectedin is an anti-cancer agent that in addition to directly targeting certain types of cancer cells, it also induces apoptosis in monocytes and macrophages. In an experimental model of prostate cancer, it was found to reduce M2-like macrophages in the marrow and skeletal metastatic tumor growth in bone (262). There is a paucity of clinical trials that investigate macrophage targeting strategies in the context of metastatic bone disease, though macrophage reprogramming in the context of efferocytosis has shown promise in preclinical studies. Uptake of apoptotic prostate cancer cells by BMDMs induced an inflammatory response that promoted bone metastatic growth but treatment with IFN-γ reprogrammed macrophages towards an M1-like state that mitigated the pro-tumoral inflammatory response (263). Chemotherapy and radiotherapy induce apoptosis in cancer cells, resulting to increased efferocytosis and subsequent suppression of inflammatory responses. Therefore, combining conventional chemotherapy and radiotherapy with an efferocytosis-targeted treatment could be a promising therapeutic approach that should be the focus of future research. While re-educating TAMs hold promise in several cancer types (260), further studies are required to fully understand its efficacy in metastatic bone diseases especially given very recent report on transcriptomically-defined “M1” macrophages associated with an aggressive cancer biology (264). Interestingly, blockade of PD-1/PD-L1, the most commonly used immune checkpoint blockage therapy in the clinic that has achieved resolution of malignancies (265), polarizes macrophage towards an M1-like phenotype (266–268) and increases TAM phagocytic potency against tumor cells (148). In addition, combining anti-PD1 therapy with metformin-loaded macrophage-derived microparticles that potently polarized TAMs from M2-like to M1-like state, boosts anti-cancer efficacy (269). Recently, PD-1 blockage was also demonstrated to have an added benefit of inhibiting osteoclastogenesis resulting in reduced bone destruction and pain (193). Of note, there are also reports indicating that TAMs might limit anti-PD-1 treatments for example through preventing CD8^+^ T cells from reaching tumor cells (147) or by removing anti-PD-1 antibodies from T cells through Fc-Fcγ receptors binding (270), though the latter can be prevented by blocking Fc/Fcγ receptor interactions (270).

### Targeting Tumor Angiogenesis

Other strategies are focused on inhibiting the tumor-promoting functions of TAMs including angiogenesis (**Figure 3**). Anti-angiogenic therapies which are largely VEGF inhibitors are used in the clinic for several cancers (271) with the first anti-angiogenic drug Bevacixumab (Avastin^®^) approved for use in 2004 by the Food and Drug Administration (FDA). While short-term relief from tumor growth is achieved in many patients, primary or acquired resistance is not uncommon and this is currently under intensive investigation (272). The existence of several novel angiogenesis regulators, such as those secreted by TAMs (181), that were not considered in the therapeutic approaches might explain the limited efficacy of current anti-angiogenic therapy. Recent studies implicate TAMs in decreased efficacy of anti-angiogenic therapy (273, 274) suggesting that targeting angiogenesis is more complex than originally thought. Findings from *in vivo* studies suggest that combinatory targeting of VEGF and other alternative effectors (275, 276) or combining anti-angiogenic drugs with TAM-targeted agents are more effective approaches (252, 277, 278), however, these are yet to be examined in metastatic bone diseases.

## Conclusion

TAMs promote skeletal metastases *via* crosstalk with tumor cells which occurs at all stages of the metastatic cascade. Therefore, whilst cure of metastatic bone diseases remains elusive, research to date strongly support that TAMs are an attractive target. Knowledge is still lacking on how they can be appropriately targeted to effectively “cure” bone metastases. Research efforts should be focused on further understanding the underlying cellular and molecular mechanisms governing TAM and cancer interactions and how these can be directed to prevent metastatic progression. Once tumor cells are established in the bone, it is clear that a cure cannot be developed until the fundamental drivers of dormancy, resistant mechanisms, reactivation and outgrowth are discovered. Although there is increasing appreciation of TAM participation in these events, further investigation of the associated complex processes is crucial. Improved understanding of TAM contribution to these events could potentially identify effective strategies that can be used to rebalance the bone microenvironment to allow conventional cancer therapies to destroy metastatic tumor cells without compromising the skeleton, ultimately curing this terminal pathology.

## Author Contributions

Both authors identified the focus and overall direction of the review. LB composed an original version of the paper and LM provided additional writing and editing. Both authors reviewed the final version. Figures were composed by LB with the assistance of LM and a graphic artist (Ken Rieger). All authors contributed to the article and approved the submitted version.

## Funding

This work was supported in part by the NIH: NCI and a University of Queensland Research Fellowship Scheme.

## Conflict of Interest

The authors declare that the research was conducted in the absence of any commercial or financial relationships that could be construed as a potential conflict of interest.

## Publisher’s Note

All claims expressed in this article are solely those of the authors and do not necessarily represent those of their affiliated organizations, or those of the publisher, the editors and the reviewers. Any product that may be evaluated in this article, or claim that may be made by its manufacturer, is not guaranteed or endorsed by the publisher.
